# Antigenotoxic Effect of Piperine in Broiler Chickens Intoxicated with Aflatoxin B1

**DOI:** 10.3390/toxins8110316

**Published:** 2016-10-31

**Authors:** Verônica da Silva Cardoso, Alane Beatriz Vermelho, Cristina Amorim Ribeiro de Lima, Jéssica Mendes de Oliveira, Marco Edilson Freire de Lima, Lúcia Helena Pinto da Silva, Glória Maria Direito, Maria das Graças Miranda Danelli

**Affiliations:** 1 BIOINOVAR—Biotecnologia, Unidade de Biocatálise, Bioprodutos e Bioenergia, Instituto de Microbiologia Paulo de Góes, Universidade Federal do Rio de Janeiro, Rio de Janeiro, RJ 21941-901, Brazil; 2 Instituto de Zootecnia, Universidade Federal Rural do Rio de Janeiro, Seropédica, RJ 23890-000, Brazil; criblima@terra.com.br; 3 Instituto de Veterinária, Universidade Federal Rural do Rio de Janeiro, Seropédica, RJ 23890-000, Brazil; jessicamop@hotmail.com (J.M.d.O.); lpinto_silva@yahoo.com (L.H.P.d.S.); gdireito@ufrrj.br (G.M.D.); danelli@ufrrj.br (M.d.G.M.D.); 4 Instituto de Ciências Exatas, Universidade Federal Rural do Rio de Janeiro, Seropédica, RJ 23890-000, Brazil; marco@ufrrj.br

**Keywords:** aflatoxin B_1_, broiler chicken, piperine, *Aspergillus*

## Abstract

Piperine is an abundant amide extracted from black pepper seeds which has been shown to have protective effects against cytotoxic and genotoxic carcinogenesis induced by certain chemical carcinogens and aflatoxin B_1_ (AFB_1_) in vitro. The aim of this work was to study, in vivo, the antigenotoxic potential of feed-added piperine on broiler chickens experimentally intoxicated with AFB_1_, using micronucleus and comet assays. The antigenotoxicity assessment of 9-day-old chicks was performed on a total of 60 chickens divided into four groups of 15 broilers each: (C) control, (P) 60 mg·piperine kg^−1^ feed, (A) 0.5 mg·AFB_1_·kg^−1^ body weight, (daily by oral route), and (P + A) co-treatment with piperine and AFB_1_. The experiment was conducted for 26 days. Chicks intoxicated with AFB_1_ showed significant genotoxic effects in the first 24 h post intoxication, and the effects remained in the other periods analyzed (48, 72, and 96 h and 26 days of treatment). The DNA damage in peripheral blood cells, the number of erythrocytes with micronuclei, and polychromatic-to-normochromatic erythrocyte ratio were significantly reduced or absent in the piperine/AFB_1_ group. No significant differences were observed between the group piperine/AFB_1_ and the control and piperine-alone groups. The addition 60 mg·kg^−1^ of piperine to the diet of the broiler chicks was safe, promoting beneficial effects in poultry health with respect to the toxic effects 0.5 mg·AFB_1_·kg^−1^ body weight.

## 1. Introduction

Aflatoxicosis is a disease resulting from the ingestion of aflatoxin in contaminated food and feed, and has become one of the most widespread and important diseases in the poultry industry [1,2,3]. Moreover, the increased human consumption of chicken has placed new demands on the poultry industry which require adaptation. Today, most consumer products are required to be safe and free of pathogens or substances that could be harmful to human health [4,5,6,7].

Aflatoxins are secondary metabolites produced by certain strains of fungi of the genus *Aspergillus*. The aflatoxins of interest to health care include aflatoxin B_1_ (AFB_1_), aflatoxin B_2_, aflatoxin G_1_, and aflatoxin G_2_ [8]. AFB_1_ is the subtype most commonly found in contaminated foods, and is considered the most toxic of the subtypes, followed by aflatoxin G_1_, G_2_, and B_2_ [9,10]. Aflatoxins are chemically stable and it is difficult to inactivate them through physical or chemical methods [9]. Consequently, when present in a feed substrate, the processing methods used to produce the feed are incapable of eliminating these toxins, or even diminishing their concentration to any significant extent [10,11,12,13,14]. 

The biotransformation of aflatoxins varies according to the species, but other factors also influence their metabolism, such as the gender, age, health, and diet of the host [10]. The mechanism of AFB_1_ biotransformation has been studied with great interest, since it is closely correlated with its toxic action [15,16]. Aflatoxin B_1_ is among the most potent of all the toxins known for their genotoxic effects, and is able to induce chromosomal aberrations, increased frequency of micronuclei development, sister chromatid exchange, unscheduled DNA synthesis, chromosome breakage, and formation of adducts in human and rodent cells [17,18,19,20,21,22,23]. 

The ever-increasing importance of plants with medicinal properties and their respective constituents in animal feed has increased the amount of research on different phytogenic compounds added either as dry or aqueous extracts, or as essential oils to the diet of animals, including broiler chickens. These additives act as performance enhancers, improving the quality of the feed for poultry [24,25].

Piperine (1-piperonyl piperidine) is an active amide present in various species of pepper, especially black pepper (*Piper nigrum* Linn.), and is responsible for the intense aroma of pepper. In addition, piperine may be found in all parts of the plant [26]. Recent studies have shown that the consumption of piperine is relatively safe, with low toxicity to cells of mammals and avian species [27,28,29,30,31]. Piperine has several biological and pharmacological properties, such as insecticidal [32], antiparasitic [33], antipyretic, analgesic, anti-inflammatory [34], and antioxidant activities [35,36]. Also, piperine has been shown to increase the bioavailability of some drugs in the body and decrease the cytotoxicity of AFB_1_ and genotoxicity in vitro without leaving residues or its derivatives in the host [37,38]. Suresh and Srinivasan [39] showed that the amount of piperine in rat tissues (0.3%) reduced significantly after 48 h, and was no longer detectable in the blood, liver, and intestine after 96 h. 

Recently, Zhang et al. [25] demonstrated the chemopreventive abilities of piperine against two osteosarcoma cell lines (HOS and U-2OS) metastasis and Greenshields et al. [40] demonstrated its protective properties against cancer cell migration in vitro through the inhibition of matrix metalloproteinase-2 and -9 expression.

The interference of piperine in the aflatoxin mechanism of action, however, has been minimally studied. Reen et al. [37] showed that piperine was able to significantly decrease the toxicity of AFB_1_ in rat hepatocytes in vitro and Selvendiran et al. [41,42] demonstrated the protective effects of piperine against cytotoxic and genotoxic carcinogenesis induced by certain chemical carcinogens and AFB_1_. Gagini et al. [43] showed that piperine treatment in intoxicated rats decreased hepatic injuries and leukocyte depletion over a 23 day-period. Previous studies by our group have demonstrated that piperine intake decreased the severity of the toxic effects of aflatoxins in broiler chickens, preventing the development of liver injury and the adverse effects on hematological parameters, which are characteristic of aflatoxicosis. These data suggest that piperine might be used to prevent the effects of aflatoxin ingestion [44]. The poultry industry seeks the development of a feed additive with a natural, non-toxic substance such as piperine not only to promote the improvement of poultry health but also to contribute to increased productivity in this industry. Piperine can be easily obtained and purified, it leaves no detectable residues in meat and meat products, and it isalso able to protect against the toxic effects of AFB_1_. Owing to the vastness of the Brazilian territory, it is still difficult to implement and enforce existing laws and regulations for the control of aflatoxins in animal feed. However, the current ceiling for aflatoxins in poultry production is 50 µg·kg^−1^ (B_1_ + B_2_ + G_1_ + G_2_) for all nutrients for chickens during the initial, developing, and final stages of growth, according to Brazilian legislation (MAPA) [45]. In Europe the limit is 20 µg·kg^−1^ [46] for aflatoxin B_1_ and in the USA the legislation determines that the concentration should not exceed 20 µg·kg^−1^, calculated as the sum of the aflatoxins B_1_ + B_2_ + G_1_ + G_2_ [47]. 

Brazil is the largest exporter and third largest producer of chicken in the world. Contamination with aflatoxins in poultry is a common problem in Brazil. Rossi et al. [48] detected aflatoxins in 72.1% of feed samples at average levels ranging from 0.0022 mg·kg^−1^ (pre-starter) to 0.0064 mg/kg (grower). Piperine potentially could be used as a neutralizing factor for aflatoxin B_1_. Reen et al. [37] showed that piperine protects rat cells in vitro by impairing the generation of toxic metabolites mediated by CYP4502B1 dependent activation of AFB_1_. This data motivates the present study. The study aimed to analyze the antigenotoxic capacity of piperine as a feed additive and its effect on the toxicity of aflatoxin B_1_ in vivo using broiler chickens (*Gallus gallus*). 

## 2. Results and Discussion

The addition of phytobiotics to chicken feed has been investigated with great interest by various researchers. Some phytobiotics act as growth promoters and others can neutralize the toxic effects of xenobiotics like AFB_1_ [43,49,50]. The covalent bonding of AFB_1_-epoxide with DNA modifies the structure of the nucleic acid and biological activity, generating RNA and DNA adducts with potential mutagenic, carcinogenic and teratogenic characteristics [51]. 

The supplementation of piperine in aflatoxin contaminated broiler diets maintained production rates in early stage, growth and total period significantly when compared to aflatoxin without piperine. Table 1 summarizes the results obtained with this comparison. No significant difference in feed conversion was observed between the piperine, piperine with aflatoxin, and negative control groups. It is interesting to note that in the first period (9 to 21 days) AFB_1_ interfered negatively in the weight gain (WG) and in the feed conversion (FC). In the second period (22 to 35 days), this was not observed. The AFB_1_ toxin acts slowly leading to irreparable losses in breeding. Often, cases go misdiagnosed, leading to irreparable losses in breeding. Usually there is a decreased ability to metabolize fats, starches and proteins, as well as apetite loss and the appearance of other metabolic disorders. However when analyzed throughout the total period (9–35 days) we observed a significant decrease in the WG and FC. This is probably a consequence of the initial stage in which the birds are more sensitive to the effects of AFB_1_.

Our group previously demonstrated several histopathological alterations in broilers intoxicated with aflatoxin. We observed megalocytosis, mononuclear cell infiltrate regions, congestion of epithelial cells of the bile duct, necrosis of biliary ducts, vacuolar degeneration, edema and diffuse vacuolation of hepatocytes. These effects were not found in animals treated with piperine [44]. Cardoso et al. [31] evaluated the effect of piperine in different concentrations (60, 120, and 180 mg·kg^−1^) for 35 consecutive days and the best and least toxic concentration was 60 mg·kg^−1^. 

**Figure 1:**
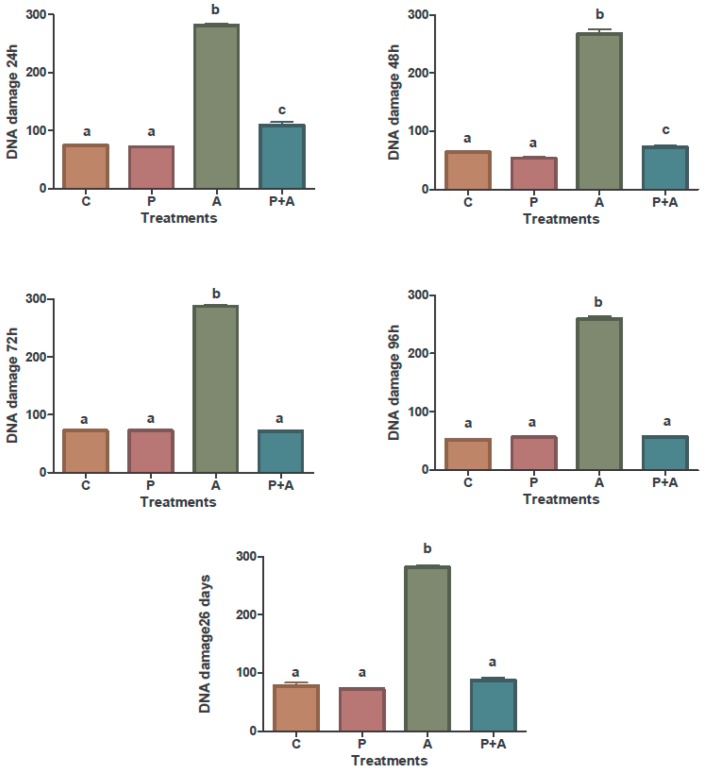
Comet assay of the action of piperine on the genotoxic effects of aflatoxin B_1_ in peripheral blood of broiler chickens. C: Control group; P: 60 ppm piperine; A: 0.5 mg·AFB_1_·kg^−1^ body weight; P + A: 60 mg piperine·kg^−1^ feed plus 0.5 mg·AFB_1_·kg^−1^ body weight; after 24, 48, 72, and 96 h, and 26 days of treatment. ^a–c^ Means and standard deviation (error bars) compared by the Student-Newman-Keuls test; means with the same letters are not statistically different (*p* > 0.05).

**Figure 2:**
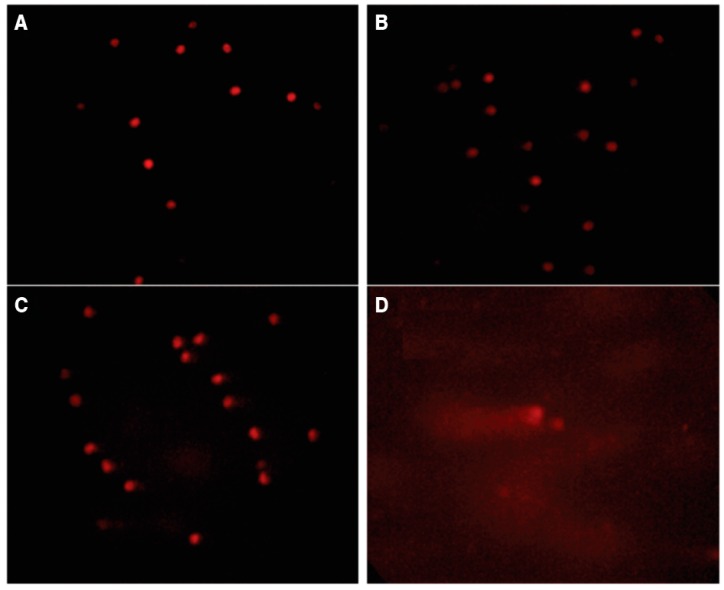
Comet assay. Damage to the DNA of peripheral blood cells was observed under an Olympus fluorescence microscope (Olympus BX41, Olympus Corporation, Tokyo, Japan) equipped with an excitation filter of 515–560 nm and a barrier filter of 590 nm (400×). One hundred cells were examined from each broiler. (**A**) Class 0, control group fed soybean vehicle carrier only; (**B**) Class 1, control group with 60 mg·piperine·kg^−1^ feed; (**C**) Class 2, group with piperine and 0.5 mg·AFB_1_·kg^−1^ body weight; (**D**) Class 3, group with aflatoxin B_1_ only.

Figure 1 represents in a graphic the percentage of DNA damage and Figure 2 showed the same damage through fluorescence microscope of peripheral blood cells in all groups. Both of the figures were developed based in the comet assay of the action of piperine on the genotoxic effects of aflatoxin B_1_.

The results demonstrated that the feed with piperine added significantly reduced the genotoxic effects induced by AFB_1_. The results of the comparative analysis between the group of broiler chickens intoxicated with AFB_1_ and the intoxicated group that received the feed with piperine added indicated that AFB_1_ causes impaired erythropoiesis and significant damage to DNA, effects that the addition of 60 mg·kg^−1^ of piperine was able to reduce or even eliminate. Such genotoxic injuries occur within a short time interval, and as such injuries could be seen 24 h post intoxication. They were still detectable at 48, 72, and 96 h and on day 26. The broiler chickens that received only piperine showed no DNA damage (comet assay) and no increase in the number of cells with micronuclei; results that were similar to the control group (*p* < 0.05). The piperine (60 mg·kg^−1^) added to the broiler feed was apparently safe, since no genotoxic effects were evident in the trials. 

Heddle et al. [52] noted that micronuclei are a short-term response to a genotoxic substance, such that its expression is dependent on the intensity of exposure to pollution and probably independent of the duration of exposure. The formation of micronuclei has been widely used in molecular epidemiology as a biomarker of chromosomal damage and genomic instability [20]. Micronuclei are fragments of chromosomes or whole chromosomes left behind during the anaphase. The micronucleus test determines genotoxic effects and can be used not only for the detection of acute genetic damage but also for chronic damage [53]. The analysis of micronuclei in peripheral blood erythrocytes is applied when there is chronic exposure to a genotoxic product [54]. In this sense, the quantification of micronuclei in normochromatic erythrocytes reports on genotoxic events that occurred in a time equal to the lifetime of the circulating erythrocytes. 

**Figure 3:**
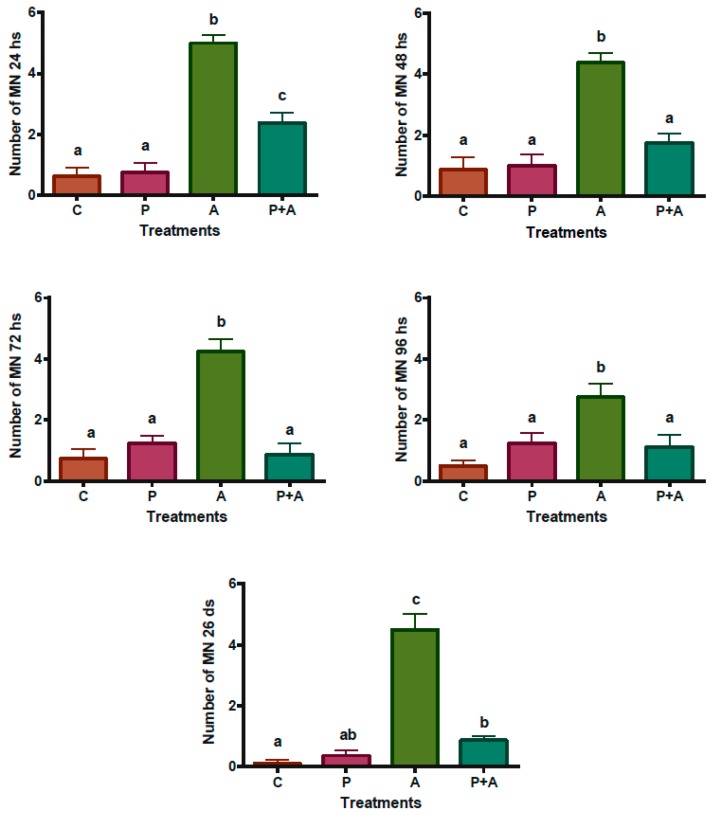
Square root of the micronuclei mean, evaluated in 2000 erythrocytes from peripheral blood samples of broiler chickens. C: Control; P: 60 mg·piperine·kg^−1^ feed; A: 0.5 mg·AFB_1_/kg body weight; P + A: 60 mg·piperine·kg^−1^ feed plus 0.5 mg·AFB_1_/kg body weight; at 24, 48, 72, and 96 h, and 26 days. Data were normalized after processing the roots. ^a–c^ Means and standard deviation (error bars) compared by the Student-Newman-Keuls test; means with the same letters are not statistically different (*p* > 0.05).

**Figure 4:**
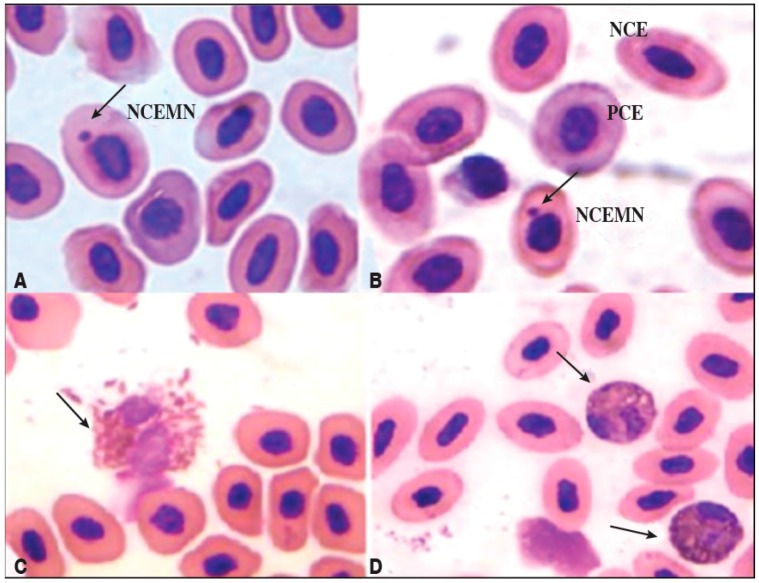
Photomicrographs of erythrocytes and heterophils. The cells were viewed under an optical microscope (Olympus^®^ CH30RF 100, Olympus Corporation, Tokyo, Japan) at a magnification of 1000×. (**A**) NCEMN: normochromatic erythrocytes with micronuclei (indicated by the arrow); PCE: polychromatic erythrocytes; (**B**) NCE: normochromatic erythrocytes, from peripheral blood of the group of chickens intoxicated with aflatoxin B_1_ (0.5 mg·AFB_1_·kg^−1^ body weight); (**C**) Severe heterophil toxicity (arrow) seen in blood smears taken from broiler chickens intoxicated with aflatoxin B_1_. (**D**) Mild heterophil toxicity (arrow) observed in blood smears of broiler chickens that received both 60 mg·piperine·kg^−1^ feed and 0.5 mg·AFB_1_·kg^−1^ body weight.

A significant increase was observed in micronuclei in normochromatic erythrocytes in the group of broilers treated with AFB_1_, indicating a chronic genotoxic effect. The treatment with piperine reduced this effect after 48 h (Figure 3). Micronuclei inside normochromatic erythrocytes were observed in Figure 4A,B. According to Reddy et al. [55], the percentage of polychromatic erythrocytes (PCEs; immature) among the erythrocytes must be determined in order to evaluate the cytotoxicity in the bone (in mammal assays). In mice, bone marrow toxicity is generally associated with the depression of spinal erythrocytes by approximately 80% or more of the control value. Although there are no published data for such an analysis in broilers, an estimation of the same calculation for broilers would give a bone marrow depression of 73.6% in Group A (receiving AFB_1_ alone), indicating a possible toxic effect on the spinal cord (Figure 4A,B). Toxic heterophils from broiler chickens intoxicated with aflatoxin B_1_ are seen in Figure 4C,D. An increase in the number of immature heterophils occurs mostly in severe bacterial infections, mycobacteriosis, aspergillosis, chlamydiosis, and severe tissue necrosis resulting from trauma or neoplasia. The presence of toxic heterophils suggests toxemia [56]. As can be observed in this work, greater quantities of toxic heterophils were present in the group receiving AFB_1_ alone (Group A) compared with the group receiving piperine (Group P) as well Group P + A (Figure 4C,D).

The group receiving AFB_1_ alone showed a significant reduction in the polychromatic-to-normochromatic erythrocyte ratio (PCE/NCE), in relation to the other groups and the time of treatment (Table 2). AFB_1_ obviously promoted the involvement of erythropoiesis, as confirmed by the reduction in the PCE/NCE ratio and the promotion of DNA damage. This effect was detected 24 h after intoxication (1st analysis). 

Any toxic effect in the bone marrow can be measured by a decrease in PCE (immature) levels, indicating a failure of production of red blood cells from the bone marrow. Therefore, the PCE level provides a fault indicator parameter of red blood cell production by the bone marrow [57].

The results of these piperine evaluations in this study show the potential for exploration in future studies. However, owing to the high coefficients of variation observed in our study, it is important to use the largest possible number of replicates per treatment. 

## 3. Conclusions

Prolonged intoxication of broilers to AFB_1_ caused lesions characteristic of aflatoxicosis, including DNA damage and bone marrow blood production errors. Daily supplementation with 60 mg·kg^−1^ of piperine in the diet of intoxicated broiler chickens ingesting 0.5 mg·AFB_1_·kg^−1^ body weight was able to reduce or even prevent the genotoxic and cytotoxic effects. Thus, piperine as a feed supplement appears to be a safe option to protect and help improve the health of broiler chickens.

## 4. Experimental Section

### 4.1. Materials

#### 4.1.1. Broiler Feed

The feed administered to the boilers was dry and made in a vertical mash mixer. First the piperine was solubilized in soy oil and then added to the feed with the other ingredients which were described by Cardoso et al. [31]. 

#### 4.1.2. Preparation of Piperine 

Piperine was obtained according to the method of Ikan [26], at a 5%–7% yield and with high purity (~98%). Gas chromatography coupled to mass spectrometry (GC-MS) showed it to have a melting temperature of 128–129 °C and spectral data identical to those reported in the literature [58]. 

#### 4.1.3. Addition of Piperine to Broiler Feed

Piperine incorporated into broiler feed was evaluated by GC-MS. Mycotoxicological analysis of the feed was performed before the experiments, according to the method of Soares and Rodriguez-Amaya [59], so that no changes in the immunological and hematological parameters could be affected by mycotoxins already present in the components of the feed (grain). The broiler feed including the corn grains used to prepare the feed contained less than the detection limits for the aflatoxin B_1_ monitored.

The composition of ingredients and nutritional requirements of the experimental chicken diet were obtained from Rostagno [60]. The piperine-containing feed was prepared in two phases: phase 1: encompassing the period between 9 and 21 days and phase 2: the beginning of the growth phase 22 to 35 days. In the initial stages (1–8 days) the feed was prepared without piperine.

#### 4.1.4. Aflatoxin B_1_

Aflatoxin B_1_ was obtained from cultures of *Aspergillus parasiticus* (CMDB 0336; origin: NRRL 2999) grown on yeast extract-sucrose agar for 11 days at 25 °C. After this period, the cultures were subjected to chloroform extraction (50 mL/plate). The extract was filtered through Whatman filter paper No. 1 and then concentrated on a rotary evaporator to give a residue. The dried extract was then diluted in 1.0 mL of chloroform and analyzed by thin-layer chromatography (TLC) on silica gel chromatography sheets (20 × 20 cm, 0.3 mm thick; Merck, Merck, Darmstadt, Germany). The extracts were separated with a mobile phase consisting of toluene/chloroform/ethyl acetate/formic acid 90% (70:50:50:20, *v*/*v*/*v*/*v*). The purified AFB_1_ was obtained by preparative TLC using silica gel (Silica Gel 60 G; Merck) on 20 × 20 cm glass chromatography plates. The mobile phase was a mixture of chloroform and acetone (90:10, *v*/*v*) for better resolution with respect to the interference present in the extracts.

The AFB_1_ was quantified by high-performance liquid chromatography (Waters ASSOC., Milford, MA, USA) equipped with a Waters pump (model 510), Rheodyne injector (Rheodyne^®^; with a 20 μL loop, Rohnert Park, CA, USA), Agilent 1100 Series fluorescence detector (Agilent Technologies, Waldbronn, Germany), UV-VIS Merck-Hitachi L-4250 detector (Merck-Hitachi, Burladingen, Germany) and D-2500 Chromato-integrator (Hitachi, Tokyo, Japan), as well as Silica 5 μ (4.6 × 150 mm) and Microsorb MV C18, 4μ (4.6 × 250 mm; Varian, Maryland, MD, USA) columns. Fluorescence was detected with excitation at 330 nm and emission at 460 nm, and compared with Ultraviolet (UV) detection at 350 nm. The mobile phases used were ethyl acetate/*n*-hexane (3:2.5, *v*/*v*) at a flow rate of 2.0 mL/min for detection by fluorescence, and methanol/water (1:1, *v*/*v*) at a flow rate of 0.7 mL/min for detection by UV. The dried extracts were quantified and kept in a freezer until administered to the broiler chickens. The AFB_1_ solutions administered to the broilers were obtained by dissolving the dry extracts with a known volume of soybean oil (solvent carrier) and homogenizing with ultrasound for 30 min at 60 °C. Aliquots of these solutions were prepared in the concentration of 0.5 mg·kg^−1^ body weight of chicken and administered daily by gavage, starting with a 10-day-old broiler. The methodology was adapted from Gagini et al. [43].

The AFB_1_ obtained from cultures was prepared using the methodology described in the Official Methods Manual of the Association of Official Analytical Chemistry (AOAC). AFB_1_ by Sigma was used as a standard solution [61]. Broilers were weighed daily during the trial so that the concentration of AFB_1_ administered satisfied the amount of 0.5 mg·kg^−1^ body weight. The AFB_1_ was administered daily to the broilers of Groups A and P + A as of 10 days old until the age of 35 days, which was the last day of the experiment.

#### 4.1.5. Chick Treatments

The experiment was conducted with 60 male chicks (9 days old) of the commercial strain Avian Cobb 500. After 9 days the intestinal tract of the broilers are physiologically mature so the broilers were adapted for 8 days before beginning the experiments [62]. The chicks were randomly divided into four groups (five broilers/cage) with three replications (3 × 5/*n* = 15) as follows: Group C were chicks that received only the vehicle (soybean oil) used in the gavage for the AFB_1_ treatment and diet without piperine; Group P comprised chicks that received only piperine (60 mg·kg^−1^ feed) added to the diet and the vehicle (soybean oil) used in the gavage daily; Group A comprised chicks treated with AFB_1_ only (0.5 mg·kg^−1^ body weight) by gavage daily and diet without piperine; and Group P + A comprised chicks treated with AFB_1_ (0.5 mg·kg^−1^ body weight) by gavage and fed a diet containing piperine (60 mg·kg^−1^ feed) daily. The aflatoxin was given to broilers which were weighed daily. The doses of piperine and AFB1 used were in accordance with Cardoso et al. [31,44].

#### 4.1.6. Broiler Performance

The following production indexes were determined at initial (9–21 days), growth (22–35 days) and total period (9–35 days of age): feed intake, weight gain and feed conversion rate from the treatments groups as described in Section 4.1.5.

#### 4.1.7. Genotoxicity Assay

Genotoxicity (based on the comet assay, presence of micronuclei, and frequency of polychromatic erythrocytes) was evaluated using blood samples collected at intervals of 24, 48, 72, and 96 h from the first day of intoxication as well as on day 26. 

The comet assay followed the protocol described by Singh et al. [63] and Tice et al. [64] with some modifications. The blood samples (1.0 mL each) were collected by ulnar vein puncture, using ethylene diamine tetra acetic acid (EDTA) as the anticoagulant. Ten milliliters of the blood sample was homogenized in 160 mL of low-melting point agarose. After homogenization, the mixture was carefully dispensed between two slides that had both been previously covered with a film of normal-melting point agarose (1.5%, *v*/*v*, in buffered saline). The slides were covered with a coverslip (60 × 25 mm) and placed in the refrigerator for 10–20 min. Thereafter, the coverslips were removed and the slides were immersed for 24 h in a vat containing a cold lysis solution (2.5 M NaCl, 100 mM EDTA, 10 mM Tris, 10% dimethyl sulfoxide, and 1% Triton X-100; pH 10). Subsequently, the slides were transferred into an electrophoresis buffer (300 mM NaOH and 1 mM EDTA, pH > 13) for 20 min at 4 °C. The electrophoresis was carried out at 25 V and 300 mA for 20 min. Then the slides were neutralized in 0.4 M Tris buffer for 5 min, fixed in absolute ethanol for 2 min, and finally stored in the dark at room temperature. For the analysis, the samples were stained with ethidium bromide (20 mL/mL) and examined under an epifluorescence microscope (Olympus BX41, Olympus Corporation, Tokyo, Japan), using a 40× objective, an excitation filter of 516–560 nm and an emission filter of 590 nm. One hundred cells were examined per animal; by calculating an average of 50 cells per slide (two slides). 

Cells were analyzed according to the integrity of the core and the length of the tail formed, as described by [64]. The nucleoids analyzed were classified according to the degree of injury, following the method of Souza and Fontanetti [65]. The comet test results was carried out by assigning numerical values (0–3) for each of the classes of harm to each individual. One hundred cells of each broiler were examined as follows: (A) Class 0, control group fed soybean vehicle carrier only; (B) Class 1, control group fed 60 ppm piperine; (C) Class 2, group with piperine and 0.5 mg aflatoxin B_1_·kg^−1^ body weight; (D) Class 3, group with aflatoxin B_1_ only.

For the micronucleus test and evaluation of the frequency of polychromatic and normochromatic erythrocytes, two peripheral blood smears of each broiler were fixed in methanol (10xx0%) for 15 min. Then the slides were washed in distilled water and stained with 5% Giemsa stain. After drying, the slides were coded and stored in boxes at room temperature. Chromosomal analysis was performed by using an optical microscope (Olympus® CH30RF 100, Olympus Corporation, Tokyo, Japan at 1000× magnification, where 1000 cells were counted per slide (two slides/broiler) [66,67]. The slides were analyzed blind for the number of polychromatic erythrocytes (PCEs), normochromatic erythrocytes (NCEs), polychromatic erythrocytes with micronuclei (MNPCEs), and normochromatic erythrocytes with micronuclei (MNNCEs). The micronuclei were identified by following the criteria adapted from the Organisation for Economic Co-Operation and Development (OECD) Test No. 474, 2014 for in vivo micronucleus tests for mammal cells [68] and by Al-Sabti and Metcalfe for fish cells [69].

At the end of the experiment (after 26 consecutive days), the broiler chickens were weighed and slaughtered in accordance with rules established by Ministry of Agriculture, Livestock and Supply, (MAPA). The experiment was carried out with 96 one-day-old male Cobb chicks housed in an experimental shed.

#### 4.1.8. Statistical Analysis

All values obtained were expressed as the mean ± standard deviation (SD). The statistical significance of different parameters between treatment groups was analyzed by analysis of variance, followed by the Student-Newman-Keuls test for comparison between means. For the micronucleus test, the calculated mean and SD for each group were analyzed using the Student-Newman-Keuls test. Statistical analysis of the comet test results was carried out by assigning numerical values (0–3) for each of the classes of harm to each individual, respectively. After obtaining the frequencies of the classes, the sum of the scores was calculated to compare the results between control group and the tests groups. The differences were considered statistically significant when the *p* value was <0.05. The software package GraphPad Prism 5.0 (version 5.0 for Windows, GraphPad Software, Inc., San Diego, CA, USA, 2010).

### 4.2. Ethical Statement

The studies involving the broiler chickens used in this study were conducted under veterinary supervision and in accordance with the normative instruction No. 3 of 17/01/2000 of the Ministry of Agriculture, Livestock and Supply, MAPA [70]. (http:/extranet.agricultura.gov.br/sislegis-consulta/consultarlegislacao.do). All experiments were approved by the Animal Care and Use Committee of Instituto de Veterinária, Universidade Federal Rural do Rio de Janeiro, Seropédica, RJ, Brasil (identification code: 23083007814, date of approval: 18 December, 2008).

## Figures and Tables

**Table 1:** Effect of piperine on: feed intake (FI), weight gain (WG) and feed conversion (FC) during the early stage (9 to 21 days), growth stage (22 to 35 days) and total period (9 to 35 days) for the broiler treatment groups.

Parameters ^1^	Treatments (ppm)				
	CONTROL	PIPERINE (P)	AFB_1_ (A)	P + A	CV *
9 to 21 days					
FI (g)	1057 ^a^ ± 36.77	1073 ^a^ ± 32.91	1059 ^a^ ± 21.63	1082 ^a^ ± 13.32	2.46
WG (g)	679.3 ^a^ ± 43.50	692.0 ^a^ ± 25.53	609.30 ^b^ ± 31.39	692.70 ^a^ ± 16.80	4.42
FC	1.55 ^a^ ± 0.05	1.54 ^a^ ± 0.02	1.74 ^b^ ± 0.06	1.56 ^a^ ± 0.02	2.38
22 to 35 days					
FI (g)	2220 ^a^ ± 75.42	2270 ^a^ ± 109.00	2339 ^a^ ± 82.39	2252 ^a^ ± 46.87	3.45
WG (g)	1359 ^a^ ± 20.98	1444 ^a^ ± 55.51	1320 ^a^ ± 68.15	1391 ^a^ ± 48.04	3.50
FC	1.63 ^a^ ± 0.05	1.57 ^a^ ± 0.05	1.77 ^b^ ± 0.06	1.61 ^a^ ± 0.02	2.80
9 to 35 days					
FI (g)	3274 ^a^ ± 98.83	3343 ^a^ ± 114.50	3365 ^a^ ± 74.73	3334 ^a^ ± 59.36	2.61
WG (g)	2039 ^a^ ± 63.54	2137 ^a^ ± 35.27	1929 ^b^ ± 37.00	2064 ^a^ ± 81.09	2.23
FC	1.60 ^a^ ± 0.05	1.56 ^a^ ± 0.04	1.71 ^b^ ± 0.02	1.61 ^a^ ± 0.04	2.66

Note: The data are expressed as mean ± standard deviation of the group of 15 animals. The averages were compared by the Student-Newman–Keuls test. ^1^ Coefficient of variation. ^a,b^ values represented by the same letter are not statistically different (*p* > 0.05). * Coefficients of variation (%). AFB_1_: aflatoxin B_1_; A: 0.5 mg·AFB_1_·kg^−1^ body weight; P: 60 ppm piperine; P + A: 60 mg piperine·kg^−1^ feed plus 0.5 mg·AFB_1_·kg^−1^ body weight.

**Table 2:** Ratio of polychromatic erythrocytes to normochromatic erythrocytes (PCE/NCE) from a total of 1000 erythrocytes of broiler chickens.

	Treatment (Mean ± SD)				
Time	CONTROL	PIPERINE (P)	AFB_1_ (A)	P + A	CV (%) *
24 h	0.097 ^a^ ± 0.027	0.081 ^a^ ± 0.024	0.045 ^b^ ± 0.011	0.070 ^a^ ± 0.016	28.14
48 h	0.107 ^a^ ± 0.029	0.089 ^a^ ± 0.010	0.060 ^b^ ± 0.017	0.096 ^a^ ± 0.018	21.57
72 h	0.130 ^a^ ± 0.036	0.126 ^a^ ± 0.027	0.075 ^b^ ± 0.071	0.116 ^a^ ± 0.024	27.10
96 h	0.153 ^a^ ± 0.036	0.149 ^a^ ± 0.038	0.084 ^b^ ± 0.025	0.156 ^a^ ± 0.021	22.99
26 days	0.268 ^a^ ± 0.020	0.263 ^a^ ± 0.024	0.062 ^b^ ± 0.018	0.246 ^a^ ± 0.017	13.68

Note: Statistical analysis of the data was done by analysis of variance with means compared by the Student-Newman-Keuls test. ^a,b^ in the rows, means followed by different letters differ statistically (*p* < 0.05). * CV: coefficient of variation; SD: standard deviation.
